# Transcriptome analysis reveals the mechanisms of flavonoid accumulation in different *Morus alba* L. varieties

**DOI:** 10.1186/s12870-025-08070-9

**Published:** 2026-01-05

**Authors:** Tao Zhou, Yue Pan, Ming Wang, Shao-li Fan, Ru-Xue Li, Lu Yang

**Affiliations:** Xinjiang Uygur Autonomous Region Academy of Forestry, Ürümqi, 830000 China

**Keywords:** *Morus Alba* L., Transcriptome, Flavonoid accumulation, Fruit development, Transcription factor (TF)

## Abstract

**Background:**

*Morus alba* L., a perennial woody plant with significant economic and ecological value, contains abundant phytochemicals, including flavonoids, which play crucial roles in plant defense and human health. Flavonoids are a diverse group of secondary metabolites with antioxidant, anti-inflammatory, and antimicrobial properties, and are widely found in plant tissues and contribute to both plant fitness and human nutrition.

**Results:**

Through comparative transcriptome analysis between Black Mulberry (HS) and Medicinal Mulberry (YS), we identified numerous differentially expressed genes (DEGs) associated with flavonoid biosynthesis. Key structural genes, including *CHS1*, *CHI*, *F3H*, *F3’H*, and ANS, as well as transcription factors (TFs) such as MYB306, BHLH106, and BHLH153, exhibited cultivar-specific expression patterns that strongly correlated with flavonoid accumulation during fruit development. Notably, the flavonoid content was consistently higher in YS than in HS, especially at later developmental stages. Furthermore, weighted gene co-expression network analysis (WGCNA) revealed a key module enriched with genes involved in flavonoid synthesis and related regulatory pathways, providing new insights into the transcriptional regulation of flavonoid accumulation in *M. alba* L.

**Conclusions:**

The study extends the understanding of flavonoid biosynthesis in plants by highlighting the cultivar-specific regulation of key biosynthetic genes and transcription factors. The results offer a theoretical basis for improving *M. alba* L. varieties and enhancing their nutritional and medicinal values. By integrating multi-omics approaches, future research can further unravel the complex regulatory networks governing flavonoid accumulation and explore their applications in agriculture, food, and medicine.

**Supplementary Information:**

The online version contains supplementary material available at 10.1186/s12870-025-08070-9.

## Background

*Morus alba* L. (mulberry) is a perennial woody plant belonging to the genus *Morus* with in the family Moraceae. It is widely distributed and holds significant economic and ecological value. Various tissues of *M. alba* L., including leaves, branches, fruits and bark, are rich in phytochemicals such as phenolic acids, flavonoids, flavonols, anthocyanins, macronutrients, vitamins, minerals, and volatile aromatic compounds, underscoring its excellent pharmacological abilities [1, 2]. Pharmacological studies have demonstrated that *M. alba* L. possesses a broad spectrum of bioactive properties, including antimicrobial, anti-inflammatory, immunomodulatory, analgesic, antipyretic, antioxidant, anticancer, antidiabetic, and protective effects on gastrointestinal, respiratory, cardiovascular, neurological, and dermatological systems, as well as hypolipidemic and anti-obesity activities [3]. Furthermore, mulberry leaves and fruits are valuable nutritional sources, providing high-quality protein, carbohydrates, dietary fiber, organic acids, vitamins, and minerals, while being low in fat [4]. Mulberry leaves have been recognized as both food and medicine and have been used to produce various functional foods and are widely used as feed in animal husbandry and aquaculture. The fruits of *M. alba* L. can be used to make jam, mulberry wine, oral liquid and other snack foods [5, 6]. Additionally, *M. alba L.* contributes to ecological restoration, wood production, and bioenergy development [7].

Flavonoids are a diverse group of secondary metabolites found in plants, are known for their antioxidant, anti-inflammatory, and antimicrobial properties [8, 9]. They play a crucial role in plant defense and contribute to the nutritional quality of many foods. As phenolic compounds, flavonoids encompass subclasses such as flavones, isoflavones, and anthocyanins. They are present in fruits, vegetables, nuts, seeds, stems, and flowers as well as tea, wine, propolis, and honey, and represent a common constituent of the human diet [8, 9]. In plants, flavonoids function as antioxidants, contribute to pest resistance, and participate in signal transduction. They act as scavengers of reactive oxygen species, including superoxide anions, hydroxyl radicals, or peroxy radicals [9]. Flavonoids are derived from the phenylpropanoid metabolic pathway and have a basic structure that comprises a C15 benzene ring structure of C6-C3-C6 [10]. Phenylalanine is converted to p-coumaroyl-CoA through the activity of phenylalanine ammonia-lyase (PAL), cinnamic acid 4-hydroxylase (C4H), and 4-coumarate: CoA ligase (4CL) [11]. Chalcone is the first key intermediate metabolite in flavonoid biosynthesis. One molecule of p-coumaroyl-CoA and three molecules of malonyl-CoA generate naringenin chalcone through the action of chalcone synthase (CHS) [12]. In addition, other key enzymes, including chalcone isomerase (CHI), Chalcone reductase (CHR), flavone synthase (FNS), flavanone-2-hydroxylase (F2H), and isoflavone reductase (IFR) also contribute to flavonoid synthesis [13–16]. Transcriptomic studies have identified several transcription factors (TFs) involved in flavonoid biosynthesis. In red-fleshed apples, the transcription factor *MdWRKY11* promotes the expression of genes such as *F3H*, *FLS*, *DFR*, *ANS*, and *UFGT*, thereby enhancing flavonoid and anthocyanin accumulation [17]. Similarly, in Tartary buckwheat, MYB TFs were shown to regulate flavonoid synthesis, where FtMYB31 TF enhancing rutin content [18]. In *Oroxylum indicum*, MYB, bHLH, and WD40 TFs are abundant and play key role in regulating flavonoid biosynthesis [19].

This study aimed to explore the molecular mechanism of flavonoid accumulation in different varieties of *M. alba* L. by performing comparative transcriptome analysis. We performed transcriptome sequencing on two *M. alba* L. cultivars to identify genes and pathways involved in flavonoid metabolism. It thereby seeks to provide a theoretical foundation for elucidating the regulatory network of flavonoid metabolism, with implications for cultivar improvement, disease resistance, resource development, and applications in medicine, food, and feed industries.

## Materials and methods

### Plant materials

The fruit and leaves of *M. alba* L. were collected from the peach and mulberry national forest germplasm resources bank in Xinhe County, Xinjiang Uygur Autonomous Region, China (latitude 41.4536, and longitude 82.6543). Mulberry trees have been identified by Jia Wei, a professor of botany at Zhejiang Academy of Agricultural Sciences. The plant variety identification samples are stored in the National Germplasm Resource Facility Xinjiang Branch Plant Variety Identification Specimen Museum (Specimen sample numbers: Black Mulberry 2015014XJ, Medicinal Mulberry 2015011XJ). For the variety characteristics and related information of black mulberry and medicinal mulberry, please refer to Dou Ziwei et al. [20]. We collected fruit in four different periods of Black Mulberry (HS) and Medicinal Mulberry (YS), including stage one (leaves spread out stage, HS-1 and YS-1), stage two (post-flowering green fruit stage, HS-2 and YS-2), stage three (enlarged green fruit stage, HS-3 and YS-3), stage four (purple fruit stage, HS-4 F and YS-4 F), and leaves were collected in stage four (HS-4 L and YS-4 L). All samples were cleaned with ultrapure water and immediately frozen in liquid nitrogen and stored at -80◦C for subsequent experiments.

### Transcriptomic analysis

Total RNA was extracted using TRIzol Reagent (Invitrogen, Carlsbad, CA, USA), and RNA integrity was evaluated using the Agilent 2100 Bioanalyzer (Agilent Technologies, Santa Clara, CA, USA). The cDNA library was constructed via synthesis of the second-strand cDNA by PCR amplification, and purification of cDNA fragments was achieved by end-repairing and adapter-connecting. RNA-seq was performed by Novaseq 6000 (Illumina, San Diego, CA, USA). The sequencing depth of each sample was 30X. The paired-end sequencing method was adopted, and the size of the library insertion fragments was 250–500 base pairs (bp). The original transcriptome data have been uploaded to the National Center for Biotechnology Information (NCBI, National Library of Medicine, Bethesda, MD., USA) database (PRJNA921738). After removing Reads containing connectors and low-quality Reads, high-quality Clean Data is obtained. The high-quality reads were aligned to the reference genome of *M. alba* L. using HISAT2 (v2.2.0) [21], a highly efficient system for aligning sequencing reads to a reference genome. The aligned reads were then assembled into transcripts and their abundance was quantified using StringTie (v2.2.0) [22], a fast and efficient assembly program. Differential gene expression analysis was performed using the DESeq2 (v1.20.0)package in R. Genes with a fold change ≥ 2 and a false discovery rate (FDR) < 0.05 were considered as differentially expressed genes (DEGs). The biological replicates were conducted three times for each sample, and each duplicate sample was sequenced by an independent library.

### Quantitative real-time PCR (qRT-PCR) analysis

The cDNA was synthesized using RNA PrimeScript™ RT reagent Kit with gDNA Eraser (Cat. No. RR047A, Takara, Tokyo, Japan). Quantitative real-time PCR (qRT-PCR) was performed using the TB Green^®^ *Premix Ex Taq*™ II FAST qPCR Kit (Cat. No. CN830A, Takara, Tokyo, Japan). All qRT-PCR analyses were performed using the following conditions: denaturation at 95 ◦C for 30 s, followed by 40 cycles of 95 ◦C for 10 s, and then at 60 ◦C for 30 s. All reactions were repeated three times in the experiments, and the 2^−∆∆Ct^ method was used to calculate the relative expression of each unigene. The *ACTIN* was used as a reference control [23]. Gene primers designed using Primer 5.0 are listed in Table S1**.**

### Data analysis

The heatmaps were drawn using the pheatmap package available in RStudio. Cluster analysis was performed via the OmicShare cloud platform (https://www.omicshare.com/, accessed on 12 November 2024). Origin 2018 was used to map the expression trend of genes.

## Results

### Statistics on the quality of transcriptome sequencing and assembly results for *M. alba* L

**Fig. 1:**
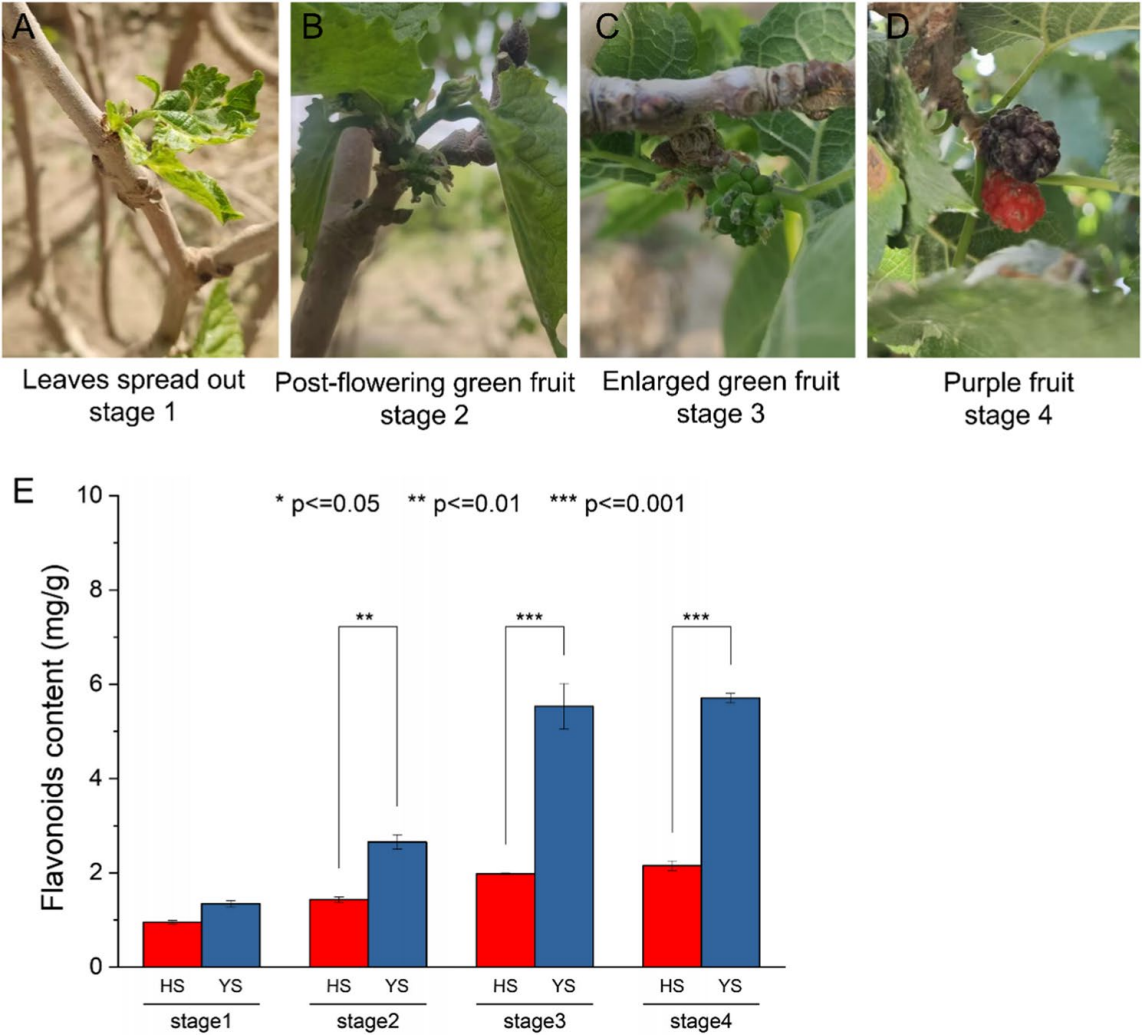
Photographs of the fruits of *M. alba* L. in different periods. **A** stage one of medicinal mulberry (leaves spread out stage, YS-1); (**B**) stage two of medicinal mulberry (post-flowering green fruit stage, YS-2); (**C**) stage three of medicinal mulberry (enlarged green fruit stage, YS-3); (**D**) stage four of medicinal mulberry (purple fruit stage, YS-4 F); (**E**) Detection of flavonoid content in different periods

Ten cDNA libraries were constructed for stage one (leaves spread out stage, HS-1 and YS-1), stage two (post-flowering green fruit stage, HS-2 and YS-2), stage three (enlarged green fruit stage, HS-3 and YS-3), stage four (purple fruit stage, HS-4 F and YS-4 F), and leaves (HS-4 L and YS-4 L) of *M. alba* L. (Fig. 1A-D). Each library contained three biological replicates, and the cDNA libraries were sequenced using the Illumina HiSeq high-throughput sequencing platform. The number of raw reads generated for each cDNA library varied between 31,933,186 and 45,237,766. The percentage of Q30 is greater than 90.51%, and the GC content was within 45.1-47.56% (Table S2). The flavonoid content of the mulberry in each stage was detected. The results showed that the flavonoid content of the mulberry was the lowest in stage 1 and increased gradually with its development, and the flavonoid content of YS was higher than that of HS in every stage, especially in stage 3 and stage 4 (Fig. 1E).

### Identification of differentially expressed genes (DEGs) between two cultivars in the fruit development process

Black Mulberry (HS) and Medicinal Mulberry (YS) are two important *M. alba* L. cultivars in Xinjiang; they all have black fruits, but their nutritional value and medicinal value are different [24]. To investigate transcriptional changes between two cultivars in the development process, we conducted a Principal Component Analysis (PCA) and observed that PC1 and PC2 were quite different between HS and YS (Fig. S1A). DEGs occurred in HS and YS at each stage including the leaves spread out stage (Fig. S1B). The DEGs were analyzed, resulting in the generation of a heatmap comprising four clusters (Fig. S1C-D).

**Fig. 2:**
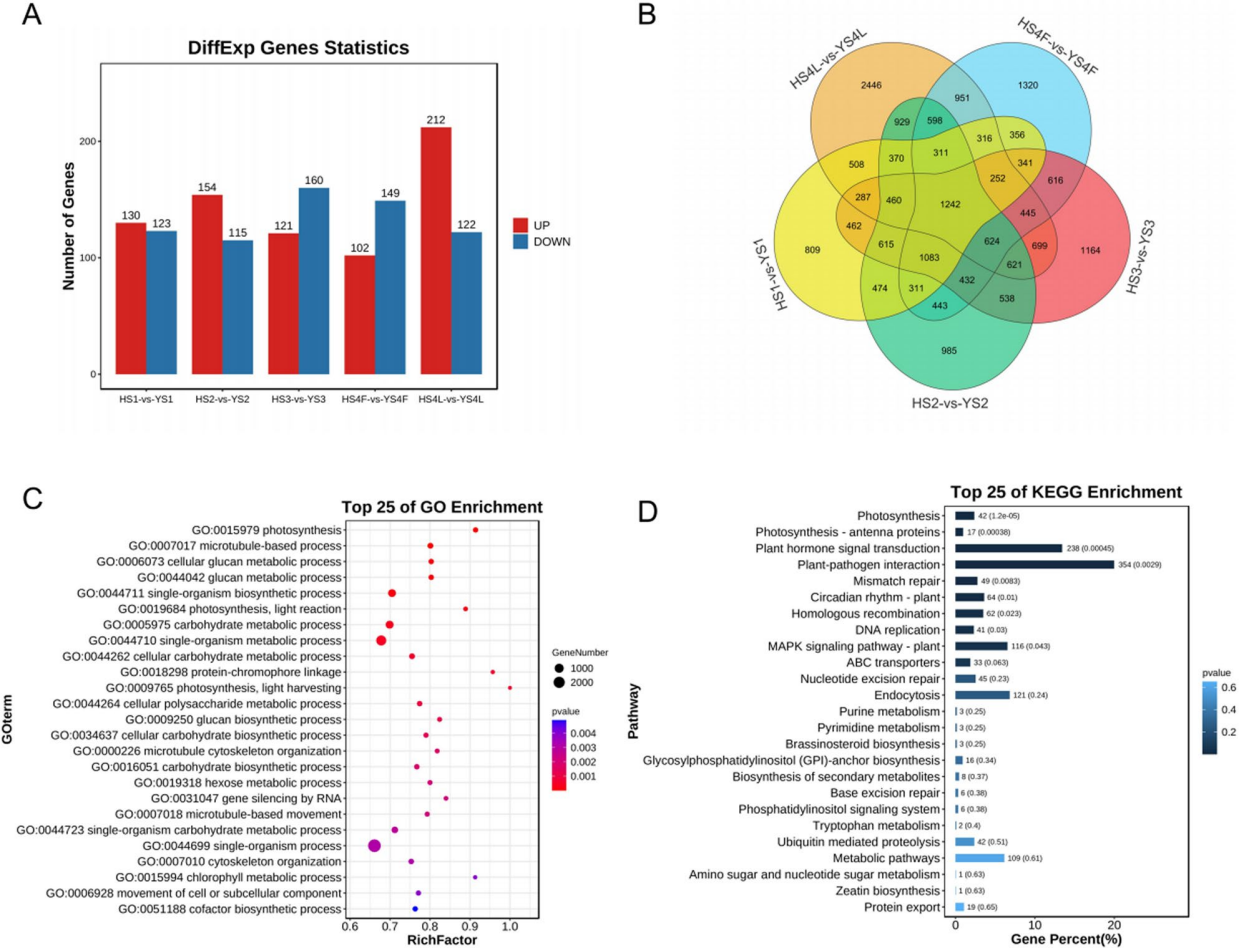
Graphical representation of analytics of DEGs specific to different developmental stages in samples of *M. alba* L. varieties in terms of GO enrichment and KEGG pathway enrichment. **A** Distribution of DEGs specific to fruit development; (**B**) Ven diagram showing distribution of the DEGs among the varieties; (**C**) Distribution of DEGs by GO enrichment; (**D**) Distribution of DEGs by KEGG pathway

To further assess the fruit ripening process transcriptional changes between HS and YS, we identified DEGs that occurred between HS and YS at each time point (Fig. 2A). In stage 3 and stage 4, there are significant differences between HS and YS; therefore, the top 50 DEGs in these stages were selected for overexpression and silencing studies (Table S3). We analyzed all DEGs, in which 1242 DEGs are included in each time point, and we analyzed the expression of these DEGs, the genes higher expressed in YS are more than HS (Fig. 2B, S2, Table S4). We conducted GO term analysis revealed that the highly expressed DEGs in YS pertained to photosynthesis, microtubule-based process, cellular glucan metabolic process, glucan metabolic process, single-organism biosynthetic process, photosynthesis, light reaction, carbohydrate metabolic process, single-organism metabolic process, cellular carbohydrate metabolic process, protein-chromophore linkage (Fig. 2C). The Kyoto Encyclopedia of Genes and Genomes (KEGG) analysis indicated that DEGs are involved in pathways that regulate photosynthesis, photosynthesis-antenna proteins, plant hormone signal transduction, plant-pathogen interaction, mismatch repair, circadian rhythm-plant, homologous recombination, DNA replication, mitogen-activated protein kinase (MAPK) signaling pathway - plant, ABC transporters, nucleotide excision repair (Fig. 2D). Thus, it became clear that there are differences between HS and YS in pattern of expression of genes involved in their development process. Although the pathway directly related to flavonoid synthesis did not appear in GO/KEGG results, the indirectly related pathways, plant hormone signal transduction, may be involved in regulating flavonoid synthesis. For instance, jasmonate-responsive gene *FtOPR is* involved in flavonoid synthesis in Tartary buckwheat [25].

### Flavonoid biosynthesis-related genes identification

**Fig. 3:**
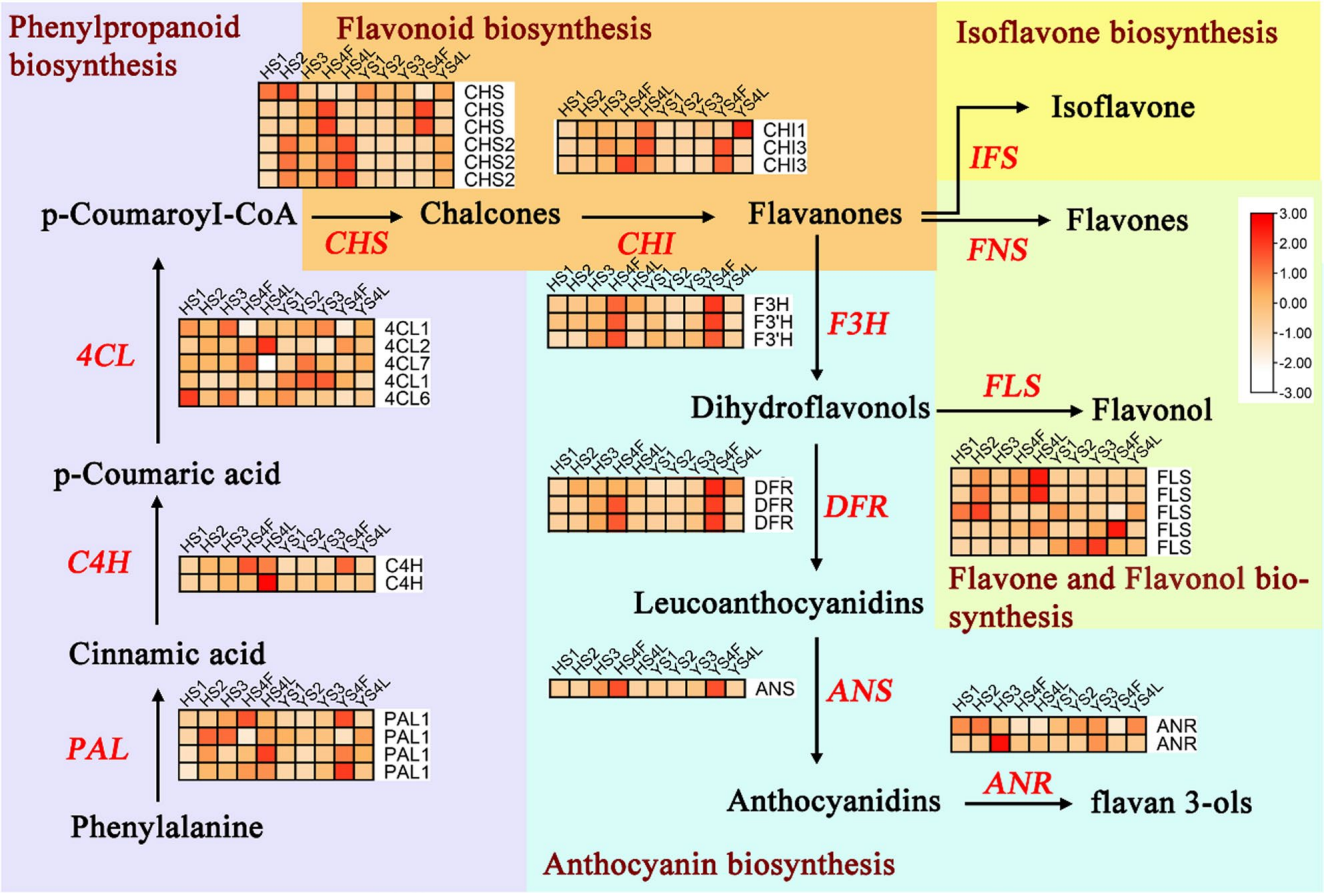
Plant flavonoids biosynthetic pathway and key enzyme gene expression heatmaps. Legend: The key enzymes of each branch are marked in red font in the figure, and the expression levels of the corresponding key enzyme genes are presented in the form of a heatmap (the darker the color, the higher the expression level, and the color scale on the right is the expression level scale)

Screening the DEGs enabled the identification of 51 key flavonoid-related structure genes. These include four PALs, two C4Hs, 13 4-coumarate-CoA ligases (four CLs), 15 CHSs, three CHIs, one flavanone 3-hydroxylase (F3H), two flavonoid 3’-hydroxylase (F3’H), three flavonol synthase (FLS), three DFRs, one anthocyanidin synthase (ANS), and two anthocyanidin reductase (ANR). The expression trends of these genes were dramatically different between the two varieties. Most of these genes exhibited relatively high expression levels at the first stage in YS compared with HS. Four *4CL* (M.alba_G689, M.alba_G4749, M.alba_G168, M.alba_G1816), one *CHS* (M.alba_G1246), one *FLS* (M.alba_G12414), and one *ANS* (M.alba_G5184) are highly expressed in YS in stages two to four and the leaf. Two *PAL* (Morus_alba_newGene_4256, Morus_alba_newGene_4255) and two *4CL* (M.alba_G787, M.alba_G135), one *CHS* (M.alba_G8924), one *CHI* (M.alba_G3475), one *F3H* (M.alba_G5697) and three *DRF* (M.alba_G13171, M.alba_G13172, M.alba_G13173) are highly expressed in the third stage in YS, while in other stage they are low expressed in YS compared with HS (Fig. 3).

### Flavonoid-related modules identification

**Fig. 4:**
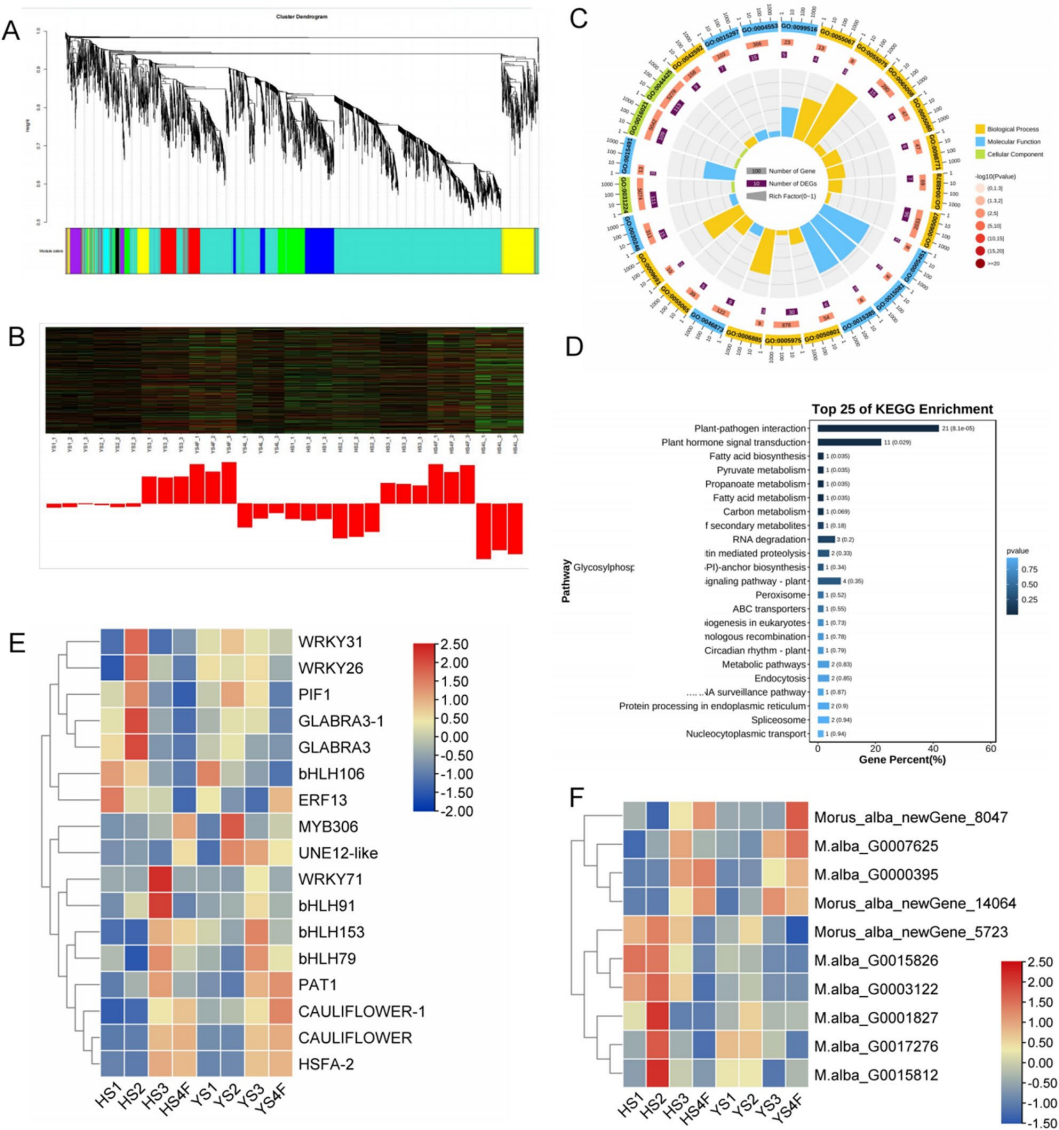
Gene cluster analysis. **A** Clustering analysis based on WGCNA, with 17 modules of different colors. **B** Heatmap of the red module in the WGCNA analysis of different samples. **C** GO enrichment analysis of genes in the red module. **D** KEGG enrichment analysis of genes in the red module. **E** Transcription factor expression heatmap. **F** The expression thermogram of flavonoid synthesis-related genes in the red module

To screen modules related to fruit development, a co-expression network was constructed using Weighted Gene Coexpression Network Analysis (WGCNA). All genes were divided into 17 modules of different colors (Fig. 4A); among them, the red module had a significant correlation with YS4F and HS4F, with 467 genes in the red module (Fig. S3, 4B, Table S5). To further understand the function of these genes, we conducted GO and KEGG analyses; these genes were significantly enriched in biological regulation and carbohydrate metabolic process, plant-pathogen interaction, and plant hormone signal transduction pathway (Fig. 4C-D). These results indicate that the genes in the red module may play an important role in fruit development, especially in regulating plant physiological processes and coping with external environmental factors (such as pathogen invasion). GO analysis helps us to understand the specific roles of these genes in cell functions and biological processes, while KEGG analysis reveals the metabolic pathways and signal transduction mechanisms they participate in. These findings provide important clues and foundations for further exploring the molecular mechanism of fruit development. In the red module, we found several TFs that may be involved in the flavonoid accumulation. MYB306 has a higher expression in YS2 than HS2, BHLH106 has a higher expression in YS1, and BHLH153 has a higher expression in YS3. Morus_alba_newGene_8047 encodes an anthocyanidin-3-glucoside rhamnosyltransferase, *M.alba*_G0007625 encodes a caffeoylshikimate esterase, which has a higher expression in YS4F, may play an important role in anthocyanin synthesis (Fig. 4E-F).

### RNA-seq validation by quantitative real-time PCR

**Fig. 5:**
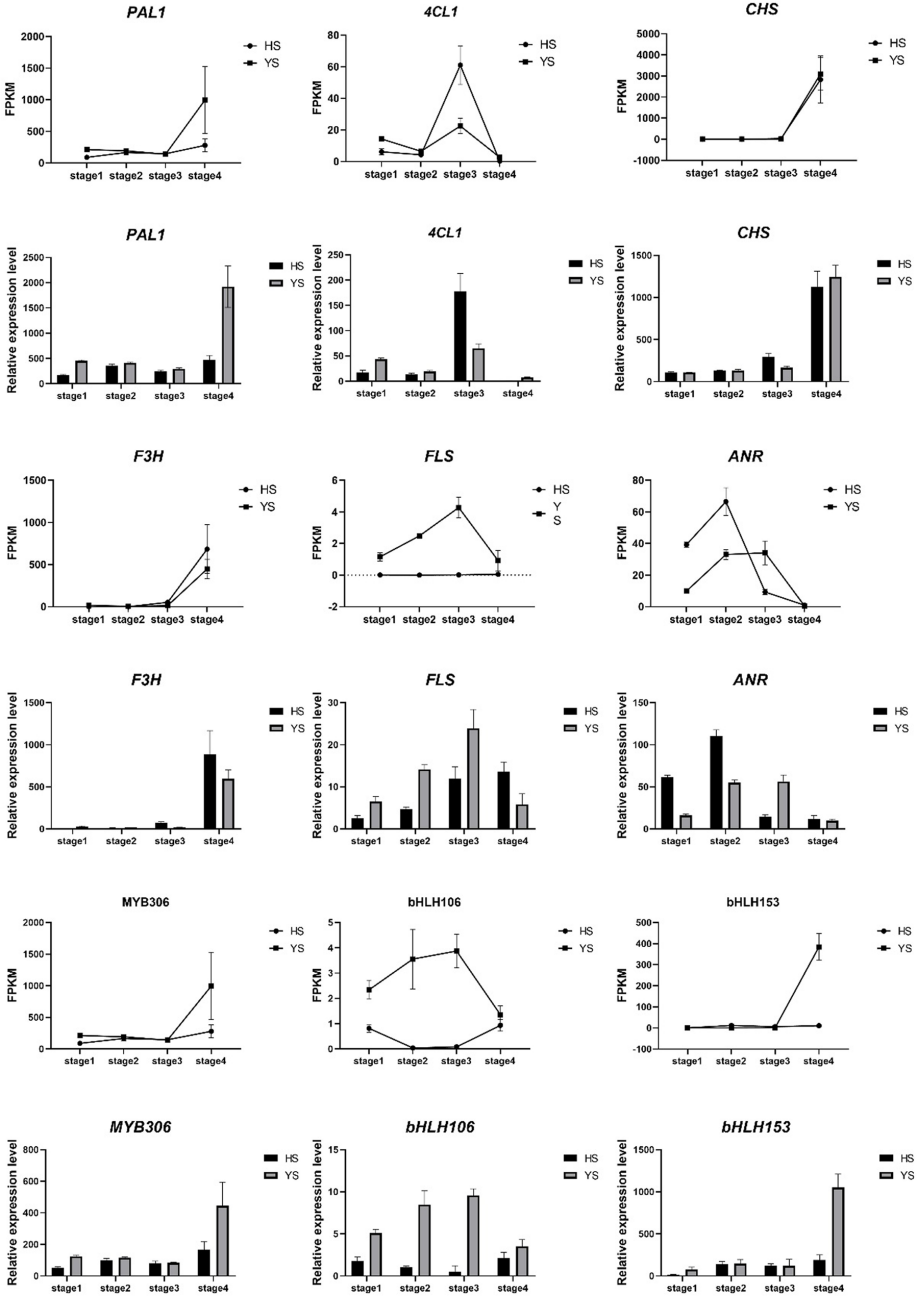
Analysis chart of transcriptome (FPKM) and qRT-PCR verification expression patterns of key genes and TFs in flavonoid biosynthesis. Legend: This graph presents the FPKM values of the transcriptome and the relative expression levels of qPCR through line graphs and bar graphs. Among them, "HS" and "YS" represent different sample groups. The figure clearly shows the expression trends of each gene at different stages and in different samples, as well as the consistency of the transcriptome and qPCR verification results

To validate the RNA-seq results, we performed quantitative real-time PCR (qRT-PCR) on selected genes. We randomly selected nine DEGs involved in flavonoid biosynthesis and TFs for qRT-PCR analysis. The qRT-PCR results showed consistent expression patterns with the RNA-seq data (Fig. 5). The expression levels of these genes in YS were generally higher than in HS, confirming the upregulation of these genes in YS during fruit development. The qRT-PCR validation further supports the reliability and accuracy of the RNA-seq data, indicating that the DEGs identified in this study are indeed differentially expressed between HS and YS during fruit development.

## Discussion

### Genes related to flavonoid biosynthesis

This study contributes novel insights that extend prior research. Our comparative transcriptome analysis of HS and YS revealed distinct gene expression profiles during fruit development. Crucially, the higher flavonoid content observed in YS, particularly in stages 3 and 4 (Fig. 1E), aligns with the coordinated upregulation of key biosynthetic genes in this cultivar. For instance, the elevated expression of early pathway genes like *PAL* and *4CL* in YS (Fig. 4) suggests an enhanced flux of carbon into the phenylpropanoid pathway, providing more precursors for downstream flavonoid synthesis. This is consistent with studies in *Rubus chingii* Hu, where the coordinated upregulation of *PAL* and *4CL* was directly correlated with increased production of ellagic acid and flavonols [26].

Furthermore, the sustained high expression of critical genes such as *CHS*, *FLS*, and *ANS* in YS during stages 2 to 4 (Fig. 4) points to a more active and prolonged biosynthesis period for flavanones, flavonols, and anthocyanins. The concurrent peak in the expression of late-stage genes like *DFR* in YS at stage 3 likely channels precursors towards anthocyanin production, coinciding with the fruit’s color change and the significant rise in total flavonoids. This pattern mirrors findings in *Litchi chinensis* and *Vitis vinifera*, where the expression of *DFR* and *ANS* is a key determinant of anthocyanin accumulation and final fruit pigmentation [27, 28]. Similarly, in apples, differential expression of flavonoid biosynthesis genes has been linked to variations in fruit color and nutritional quality [17, 29]. In *Fragaria vesca*, the expression of *CHI*, *C4H*, *CHS*, *CHI*, *F3H*, *DFR*, *ANS*, and *FLS* genes has been linked to variations in flavonol content and antioxidant activity [30, 31]. Therefore, the transcriptomic differences between HS and YS are not merely quantitative but reflect a fundamentally different temporal regulation of the flavonoid pathway, which directly underpins the divergence in their nutritional and medicinal properties.

### Transcript factors related to flavonoid biosynthesis

Co-expression network analysis identified flavonoid-related TFs, with the red module showing a significant correlation with fruit development stages. GO and KEGG analyses of the red module genes revealed their roles in biological regulation, carbohydrate metabolism, and plant-pathogen interactions, underscoring their importance in the development and metabolic processes of *M. alba* L. fruits. TFs such as MYB, bHLH, and WRKY have been shown to regulate flavonoid biosynthesis, stress responses, and fruit development [32]. For example, in *Arabidopsis*, the MYB-bHLH-WD40 complex has been shown to regulate the expression of flavonoid biosynthesis genes [33, 34]. Interestingly, transient expression analyses in *A. thaliana* cells suggested that MYBL2 interacts with MBW complexes in planta and directly modulates the expression of flavonoid target genes [35]. In *Malus domestica*, the transcription factor MdWRKY11 has been shown to promote the expression of flavonoid biosynthesis genes, leading to increased flavonoid and anthocyanin accumulation [17]. Similarly, in *Oroxylum indicum*, MYB and bHLH TFs have been implicated in the regulation of flavonoid biosynthesis and stress responses [19]. Our findings in *M. alba* L. suggest that similar regulatory networks may be at play, with MYB306, BHLH106, and BHLH153 TFs playing a key role in modulating flavonoid biosynthesis during fruit development and flavonoid synthesis. The early expression peak of BHLH106 TF in YS1, YS2, and YS3 could be instrumental in initiating the flavonoid pathway, potentially by activating early biosynthetic genes like *CHS*. Subsequently, the high expression of MYB306 and BHLH153 TFs in YS4 may form a regulatory cascade that fine-tunes the pathway, promoting the synthesis of specific flavonoid sub-classes during mid to late development. This coordinated TF activity likely explains the sustained high expression of key structural genes in YS compared to HS, further promoting the accumulation of flavonoids in the later stage.

## Conclusion

In conclusion, this study elucidates key molecular mechanisms underlying flavonoid biosynthesis and fruit development in *M. alba* L. By identifying DEGs, key flavonoid biosynthesis genes, and flavonoid-related TFs, this research contributes to the elucidation of the regulatory networks controlling flavonoid accumulation in *M. alba* L. However, the study has certain limitations, such as the need for functional validation of identified genes and the inclusion of a broader range of cultivars and environmental conditions. Future research should focus on functional studies, metabolomic analyses, and expanding the sample range to further unravel the complex regulatory mechanisms of fruit development in *M. alba* L. Additionally, integrating multi-omics approaches, such as proteomics and metabolomics, could provide a more comprehensive understanding of the molecular basis of flavonoid biosynthesis and its impact on the nutritional and medicinal properties of *M. alba* L.

## Supplementary Information

Supplementary Material 1.

Supplementary Material 2.

## Data Availability

The datasets generated and analyzed in this study are available in the NCBI GenBank (PRJNA921738).
