# Supplementary material for: Transcriptome analysis reveals the mechanisms of flavonoid accumulation in different Morus alba L. varieties
Source: BMC Plant Biol. 2026 Jan 5;26:210. doi: 10.1186/s12870-025-08070-9 (PMC12871004; doi:10.1186/s12870-025-08070-9)

**Fig. S1 Graphical representation of analytics of DEGs in different samples.**


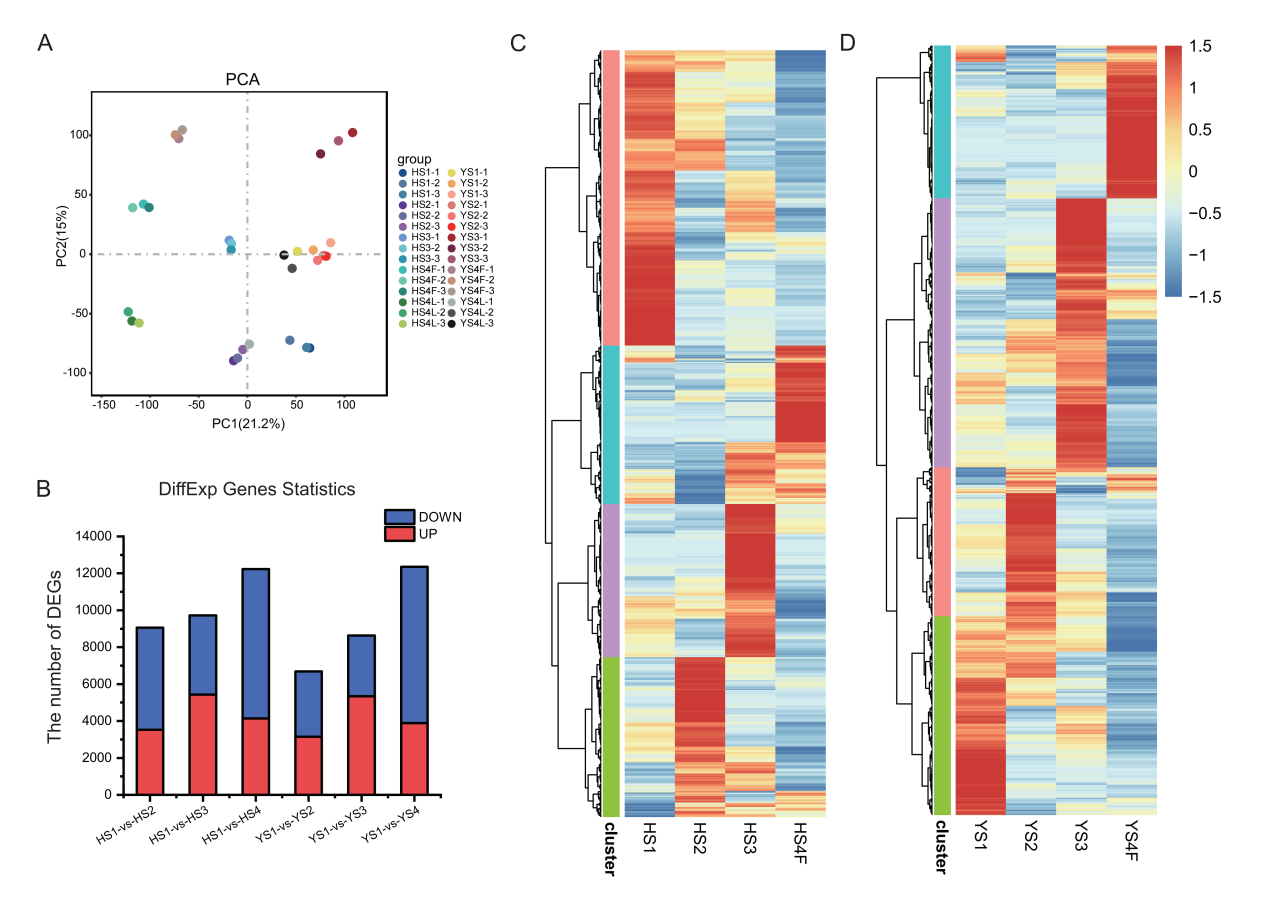


Fig. S1 Graphical representation of analytics of DEGs in different samples. (A), PCA scatter plot of DEGs; (B), Bar diagram showing global frequency distribution of DEGs; (C), Clustered heatmap of DEGs in HS samples; (D), Clustered heatmap of DEGs in YS samples.

**Figure S2 Clustered heatmap of the DEGs in HS_vs_YS**

**
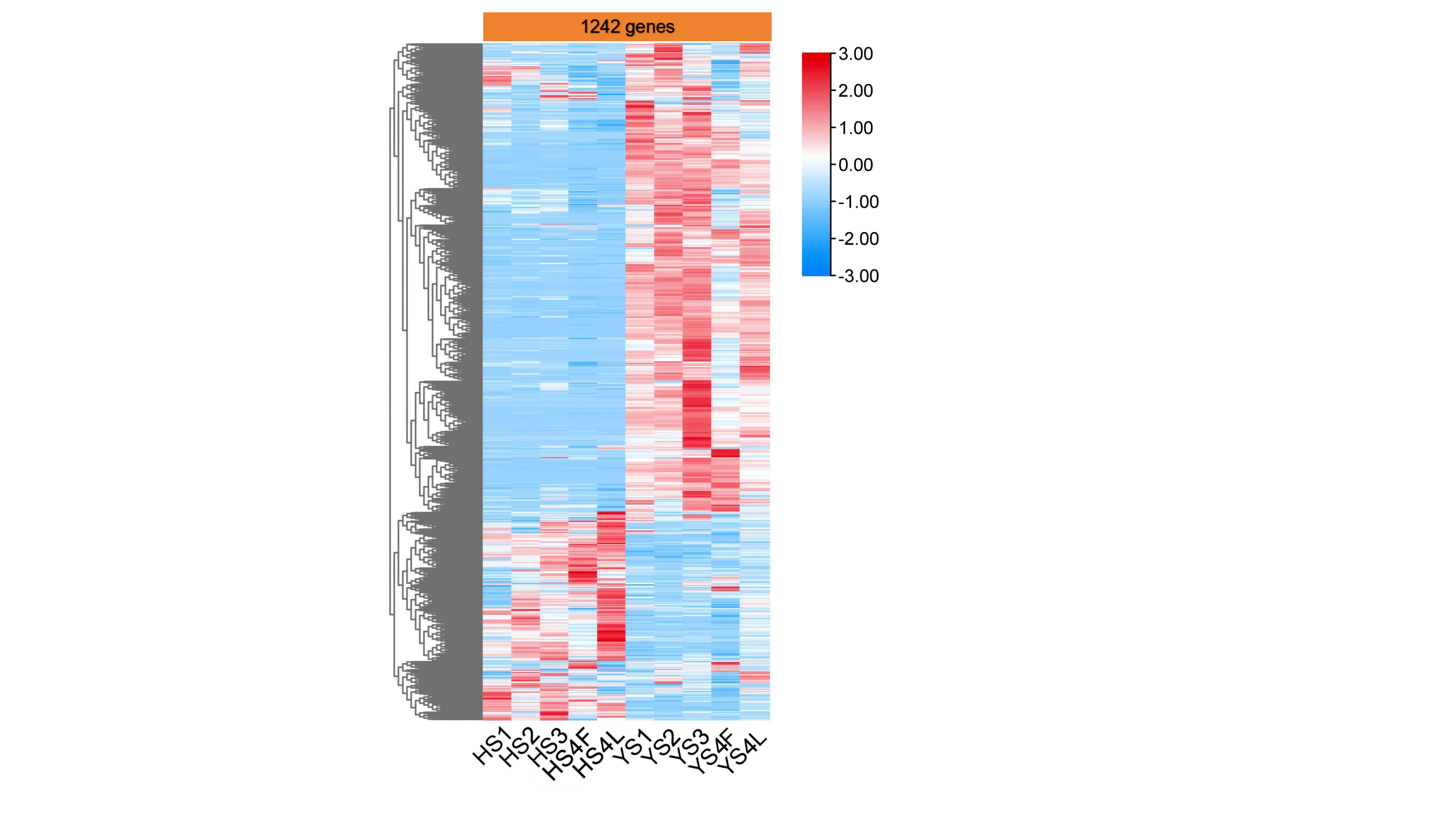
**

**Figure S3 Correlation analysis between modules and different samples.**


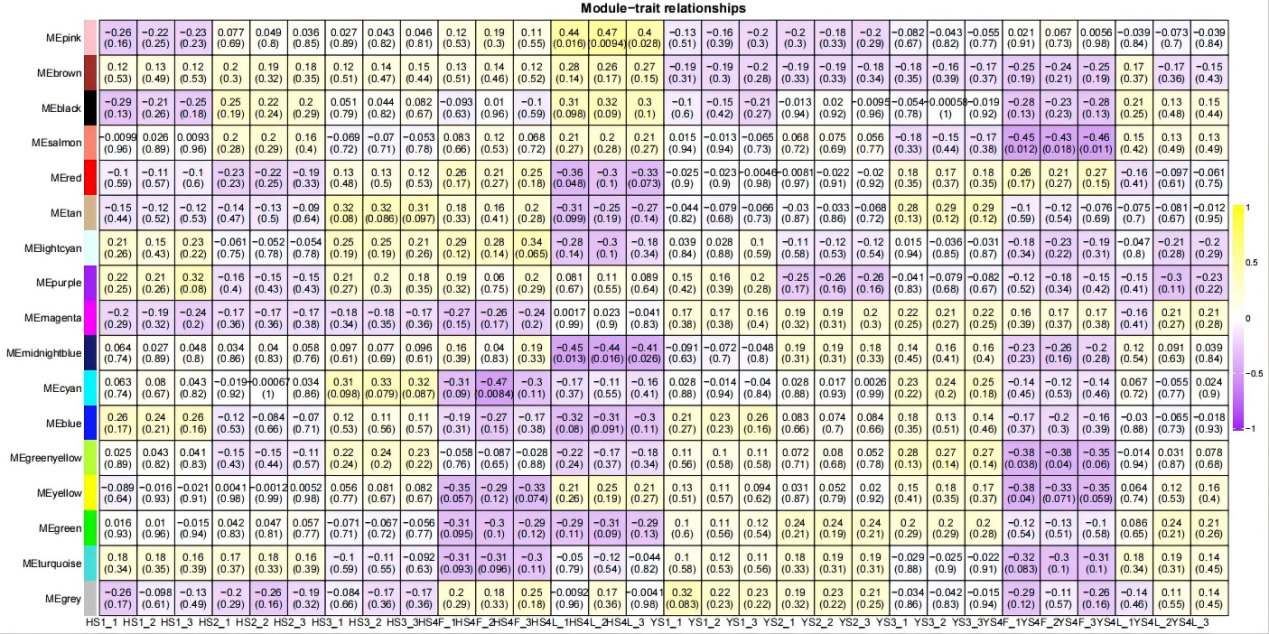

Supplement: Supplementary file 1 — Supplementary Material 1. [file 12870_2025_8070_MOESM1_ESM.docx]
